# CRMP-5-IgG Antibody: role in the bilateral uveitis with swollen disc

**Published:** 2020

**Authors:** Laura Hernández-Bel, Francisco Puchades-Gimeno, Amaya Fernandez-Diaz, Lucía Mata-Moret, Emma Beltrán-Catalán, María Luísa Hernandez-Garfella, Enrique Cervera-Taulet

**Affiliations:** * Hospital General Valencia, València, Spain; ** Hospital del Mar, Barcelona, Spain

**Keywords:** paraneoplastic conditions, optic neuritis, CRMP-5 IgG, small cell lung carcinoma

## Abstract

Autoimmunity against collapsin response-mediator protein-5 (anti-CRMP-5) has been associated with ocular inflammation in paraneoplastic syndrome.

We present a 59-year-old Caucasian man with optic neuritis and vitreous cells in both eyes (OU), at different stages. Despite the fact that the patient did not have any systemic disease, we suspected a paraneoplastic syndrome and requested CRMP-5-IgG and a mediastinoscopy. After performing the tests, a small cell lung carcinoma was diagnosed.

Autoantibody CRMP-5-IgG positivity and optic neuritis combined with vitreous inflammation was defined as a paraneoplastic entity, avoiding vitreous biopsy and allowing us to suspect malignancy before systemic symptoms appeared.

## Introduction

Uveitis is a vision threatening disease that represents a diagnostic and therapeutic challenge for ophthalmologists [**1**]. Noninfectious uveitis’ effect on years of vision loss and its economic repercussions are particularly important [**2**]. Posterior uveitis can be classified by its etiology and that includes infectious causes (tuberculosis, syphilis) and non-infectious causes (sympathetic ophthalmia, masquerade neoplastic, Behçet disease, sarcoidosis or Voght-Koyanagi-Harada disease). 

Paraneoplastic syndromes result from immune-mediated reactions produced by antibodies that cause cross-reactions between components of the tumor and other components of our body. They can be associated with many malignancies but the most common is small-cell lung cancer (SCLC) (9% of the patients) and usually precedes the tumor diagnosis [**3**].

Ophthalmic paraneoplastic syndromes impair about 0.01% to 1% of the patients with malignancies [**4**] with manifestations purely affecting the eye - retina or choroid involvement - or also involving central and peripheral nervous system. The ophthalmologist may have an important role in the work-up, because paraneoplastic syndrome can be the first sign of a non-diagnosed cancer. Bussat et al. have recently reviewed the predominant antibodies in paraneoplastic syndromes affecting the eye, identifying 9 of them: Anti-Ri, Anti-Ma, Anti-Hu, Anti-Yo (PCA1), Anti-TR (PCA2), CV-2 (CRPM5), Amphiphysin, Recoverin and Anti-surface antigen Ab VGCC [**5**].

SCLC generates from neuroendocrine-cell precursors and distinguishes itself by its fast growth and its chemotherapy and radiotherapy’s high response rates, whereas resistance to treatment is very low. In the Western world, the ratio of population with SCLC has reduced to 10-15% of the total of lung cancer cases. Most of those patients suffering from SCLC share a history of tobacco use, this being one of the main risk factors known (ESMO). A wide diversity of paraneoplastic syndromes has been related to SCLC. In fact, this cancer type has a high mutational burden, which is associated with the presence of multiple neoantigens that can modulate the immune system. 

We present the case of a patient with posterior uveitis and bilateral papillitis manifesting as a paraneoplastic syndrome, with a positive CRMP-5 antibody, comparing it with the ones in published literature. 

## Case report

A 59-year-old man consulted our service due to a bilateral and painless visual loss. He started presenting blurred vision on his left eye (LE) the prior week, which became bilateral in 48 hours. He was a smoker of 52 packets/ year and he also reported high blood pressure, which was controlled with torasemide 5mg/ day and amlodipine 10mg/ day. He denied any other relevant medical history past. 

**Fig. 1:**
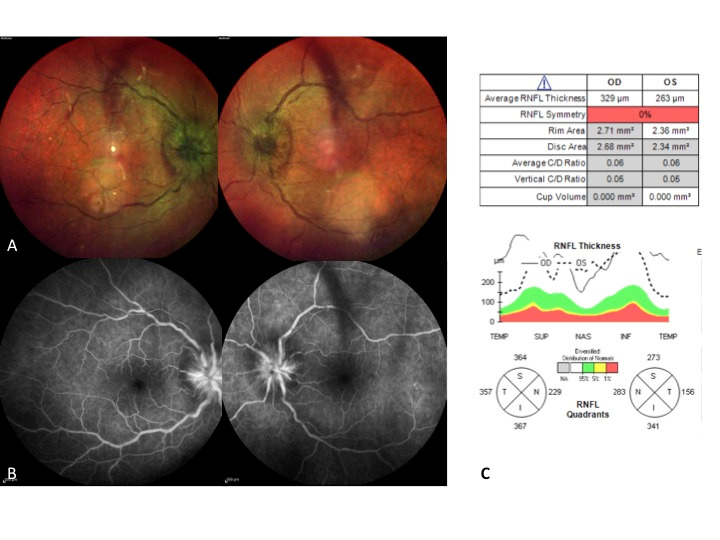
The swollen disc at diagnosis. A bilateral leakage with no signs of vasculitis is shown in the fluorescein angiography

Ophthalmic examination revealed a reduced visual acuity (VA), 20/ 125 in his right eye (RE) and hand movement in his left eye (LE). The slit lamp examination revealed a non-swollen anterior pole, but in the fundus examination we found a moderate vitreous cellularity (1+) on his RE and a dense vitreous cellularity (2+) on his LE. Moreover, we found a bilateral swollen optic disc (**Fig. 1A**), an arteriolar narrowing and vascular tortuosity without sheathing retinal veins. Fluorescein angiography examination revealed a hyper fluorescence and leakage in the optic nerve without any vasculitis signs (**Fig. 1B**). A CT-scan was normal and the chest X-ray revealed enlargement of both hila. With a suspicion of posterior uveitis with bilateral papillitis we initiated 1-gram Methylprednisolone x 5 days.

**Fig. 2:**
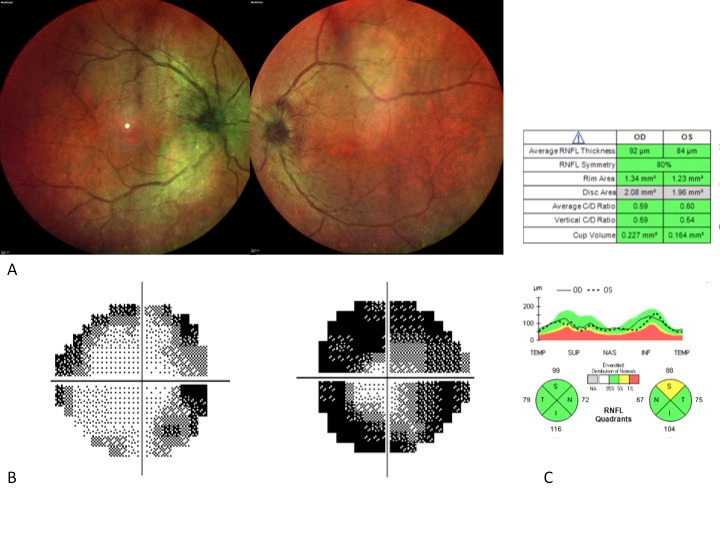
Improvement of the bilateral swollen disc after corticosteroid treatment

A total-body CT-scan only revealed enlargement of para tracheal bilateral nodes, and the cerebral MRI was normal. The purified protein derivative (PPD) skin test, Interferon-γ release assay, HLAB27, HLA B51 and Angiotensin-converting enzyme (ACE) were negative, with the rest of the biochemistry being unremarkable. Two different biopsies made by echo-endoscopy revealed necrotic areas without granulomas or evidence of malignancy. A new ophthalmologic examination showed a total resolution of the vitritis, without evidence of retinitis or choroiditis, with diminution of the swollen optic disc (**Fig. 2A**) and a fast recovery of his VA becoming 20/ 20 in OU during the first weeks, although an enlargement of the blind spot, and some peripheral scotoma in his visual field persisted (**Fig. 2C**). 

A CT-scan was repeated and multiple node enlargements in mediastinum and bilateral hilum were still identifiable, so we decided to perform a mediastinoscopy, which revealed a SCLC. Serologic evaluation of paraneoplastic autoantibody profile was positive to CRMP-5-IgG positive.

The patient was referred to the oncology department with a diagnosis of SCLC with limited involvement of the thorax. Initially, a concomitant treatment with cisplatin and etoposide together with radiotherapy was considered. However, due to the fact that all the affected territories could not be included in a single radiation field, an induction chemotherapy was started. After three cycles of treatment, a partial response was achieved, hence concomitant radiotherapy was at that point feasible. After three cycles of chemo radiation, a complete response was achieved. 

## Discussion

Acute or subacute visual loss with papillitis and vitritis is a challenging clinical problem for ophthalmologists and other clinicians. Paraneoplastic syndromes have always intrigued neurologists because of their role in the identification of the malignancy of a neurologic syndrome and in the molecular biology, which characterizes the antigens recognized by paraneoplastic antibodies, as well as in the identification of neuronal restricted proteins that are major for a successful operation of the nervous system. 

CRMP-5 is the 62kDa member of a family of nervous system-restricted proteins that mediate signaling by the axon repulsive guidance cue collapsing-1 (Sema3A) [**6**]. Autopsy findings in CRMP-5 positive patients proved a prevailing infiltration of CD8+ T cell and corresponding areas of nerve fiber and myelin loss, microglial activation with perivascular lymphocytic cuffing (90% CD8+ T cells) in mesial temporal structures, cerebellum, brainstem and spinal cord, and loss of myelinated axons in peripheral nerves, spinal rootlets and spinal sensory ganglia [**7**]. Malik et al. first reported CRMP-5 in 1992 and Honnorat et al. called it CV-1 in 1996 [**8**]. Yu et al. studied 121 CRMP-5 positive patients among 68.000 who were suspected of paraneoplastic syndrome. The most frequent diagnosis was chorea (11%) and cranial neuropathy (17%, including 10% loss of olfaction/ taste, 7% optic neuropathy). Other signs were peripheral neuropathy (47%), autonomic neuropathy (31%), cerebellar ataxia (26%), subacute dementia (25%), and neuromuscular junction disorders (12%). Lung carcinoma (mostly limited small-cell) was found in 77% of the patients; thymoma was found in 6% [**9**]. The main studies relating optic neuritis and CRMP-5-IgG, and paraneoplastic syndrome are summarized in **Table 1**. 

**Table 1:** Reports of other paraneoplastic syndromes

Table 1	Age Sex	Tumor	Presentation	Visual field (VF)	Fundoscopy	Fluorescein angiography	Evolution
Cross et al. 16 cases [**7**]	52-74 (W)	11 SCLC, 1 Breast C, 1 MGUS, 1 Mesenchyme	15 Subacute and painless, 1 progressive myelopathy	Blind spots enlargement, Arcuate, altitudinal defects, Paracentral scotoma, Peripheral constriction, General depression	Bilateral swelling (15/ 16), Cells in posterior vitreous (9/ 16), Cells in anterior chamber (1/ 16)	Nerve head hyperfluorescence, Leakage (Peripheral retinal inflammation)	6 Died (> 5 years), 8 alive (12 months), 2 no follow-up
Toribio-garcia et al. [**10**]	64 (M)		Subacute painless Bilateral, RE 0,4, LE: Hands movement		Vitritis		Resolution visual abnormalities. Not reported
Murakami Y et al. [**11**]	55 (W)	Lung adeno-carcinoma	Subacute, Bilateral, 20/ 400	Central scotoma, Enlarged blind spot	Bilateral Swelling, No inflammatory cells	Hyperfluorescence, Leakage	
Cassewell et al. [**12**]	52 M	SCLC	Painless loss of vision progressing over 4 months in OU	Constricted bilaterally with central scotomata	6/ 60 RE, 3/ 60 LE pale and swollen (**Fig. 1A**) and clinically diffuse odd retinal sheen	Optic disc diffuse leakage, OCT revealed bilateral macular atrophy, with disruption of IS/ OS junction	Chemotherapy completed. Remission
Cassewell et al. [**12**]	58 (M)	SMLC	6/ 60 RE and counting fingers LE	RE VF markedly constricted; LE VF constricted and with a dense central scotoma	OU moderate vitritis. Swollen and pale optic discs	Diffuse optic disc’s capillaries leakage and marked peripheral vessels leakage	The patient died soon afterwards
Saito M et al. [**13**]	67 (M)	SMLC	Central vision loss RE progressin over a month, VA was 0.8 RE and 1.2 LE		++ anterior vitreous cells OU. Swollen optic disc surrounded by serous retinal detachment (SRD), dilated tortuous veins OU, and subretinal opaque exudation at the fovea RE	Hyperfluorescence with late leakage from the optic disc and retinal venous wall staining OU, Hyperfluorescence at the fovea, OCT showed SRD adjacent to the optic disc OU and dome-shaped hyperreflective lesion extending from the inner nuclear layer to the photoreceptor layer (foveal exudation)	
Margolin E et al. [**14**]	62 (M)	SCLC	Slow progressive visual loss, 20/ 70 in the RE and finger counting in the LE	A mean deviation of 15 dB in the RE and 15.8 dB in the LE without localizable features	++ vitritis and bilateral papillitis	Mild late optic disc leakage in OU	
Morita M et al. [**15**]	60 (M)	SCLC	Photophobia, vision decreased and paresthesia of limbs	Reduced VA (RE 20/100, LE 20/ 22)	Few abnormal findings in the fundoscopy	No other abnormal optic nerves findings by fundoscopy and fluorescent fundus angiography	Died 7 months after diagnosis

Our case was relevant because in the diagnosis of posterior uveitis with bilateral papillitis, the CRMP-5 antibody should have been performed and malignancy should have been ruled out in order to obtain a definitive diagnosis as soon as possible. Despite the great efforts to improve the outcome of the patients with SCLC, the treatment has not changed in the past 30 years. Platinum salts and radiotherapy remain the standard of treatment in both, advanced and limited disease of the chest. The stage of the disease is the main prognostic factor, but in general, this cancer type has poor prognosis, showing an overall survival at two years of around 20% in limited-disease and < 5% in extensive-disease.

**Ethical approval**

This report has been approved by Valencia University research ethics committee and adheres to the tenents of the Declaration of Helsinki. 

**Financial support**

None.

**Conflicts of interest and source of funding**

None.

**Acknowledgements**

None.
